# Netrin-1 acts as a non-canonical angiogenic factor produced by human Wharton’s jelly mesenchymal stem cells (WJ-MSC)

**DOI:** 10.1186/s13287-017-0494-5

**Published:** 2017-02-28

**Authors:** Catalina P. Prieto, María Carolina Ortiz, Andrea Villanueva, Cynthia Villarroel, Sandra S. Edwards, Matías Elliott, José Lattus, Sócrates Aedo, Daniel Meza, Pablo Lois, Verónica Palma

**Affiliations:** 1 Laboratory of Stem Cells and Developmental Biology, Faculty of Sciences, University of Chile, Santiago de Chile, Chile; 2 Campus Oriente, Department of Obstetrics and Gynecology, Faculty of Medicine, University of Chile, Santiago de Chile, Chile

**Keywords:** WJ-MSC, Angiogenesis, Netrin-1, Tissue repair, CAM assay, HUVEC

## Abstract

**Background:**

Angiogenesis, the process in which new blood vessels are formed from preexisting ones, is highly dependent on the presence of classical angiogenic factors. Recent evidence suggests that axonal guidance proteins and their receptors can also act as angiogenic regulators. Netrin, a family of laminin-like proteins, specifically Netrin-1 and 4, act via DCC/Neogenin-1 and UNC5 class of receptors to promote or inhibit angiogenesis, depending on the physiological context.

**Methods:**

Mesenchymal stem cells secrete a broad set of classical angiogenic factors. However, little is known about the expression of non-canonical angiogenic factors such as Netrin-1. The aim was to characterize the possible secretion of Netrin ligands by Wharton’s jelly-derived mesenchymal stem cells (WJ-MSC). We evaluated if Netrin-1 presence in the conditioned media from these cells was capable of inducing angiogenesis both in vitro and in vivo, using human umbilical vein endothelial cells (HUVEC) and chicken chorioallantoic membrane (CAM), respectively. In addition, we investigated if the RhoA/ROCK pathway is responsible for the integration of Netrin signaling to control vessel formation.

**Results:**

The paracrine angiogenic effect of the WJ-MSC-conditioned media is mediated at least in part by Netrin-1 given that pharmacological blockage of Netrin-1 in WJ-MSC resulted in diminished angiogenesis on HUVEC. When HUVEC were stimulated with exogenous Netrin-1 assayed at physiological concentrations (10–200 ng/mL), endothelial vascular migration occurred in a concentration-dependent manner. In line with our determination of Netrin-1 present in WJ-MSC-conditioned media we were able to obtain endothelial tubule formation even in the pg/mL range. Through CAM assays we validated that WJ-MSC-secreted Netrin-1 promotes an increased angiogenesis in vivo. Netrin-1, secreted by WJ-MSC, might mediate its angiogenic effect through specific cell surface receptors on the endothelium, such as UNC5b and/or integrin α6β1, expressed in HUVEC. However, the angiogenic response of Netrin-1 seems not to be mediated through the RhoA/ROCK pathway.

**Conclusions:**

Thus, here we show that stromal production of Netrin-1 is a critical component of the vascular regulatory machinery. This signaling event may have deep implications in the modulation of several processes related to a number of diseases where angiogenesis plays a key role in vascular homeostasis.

**Electronic supplementary material:**

The online version of this article (doi:10.1186/s13287-017-0494-5) contains supplementary material, which is available to authorized users.

## Background

Vascular and neuronal networks share many developmental characteristics: both are ramified systems, have similar tissue distribution patterns, and during development, cellular precursors follow similar molecular cues. Due to these similarities, researchers suggest that the formation of both networks may be regulated by common factors [1]. Additionally, vascular and neuronal pathways share several guidance factors and receptors, which play a non-canonical role to control the angiogenic process. Of particular interest is the Netrin protein family, including isoforms 1, 2, 3, and 4, which role has been well characterized in the nervous system [2]. More recently, these proteins and receptors have been implicated in vascular biology [3, 4]. However, since little is known about the nature of Netrin-secreting cells, the putative endothelial receptors mediating Netrin-driven angiogenesis and especially due to the existence of conflicting reports, researchers remain puzzled about the role of the Netrin proteins in vascular development and pathological conditions [2, 5].

Netrin-1, the most characterized ligand of the family, is a critical axonal guidance protein [6] during embryonic development [7] and morphogenesis [8], and recently has been related to angiogenic processes [9]. Netrins axonal functions have been linked to two classes of receptors: (1) the deleted in colorectal cancer (DCC) family, including DCC and its orthologue Neogenin-1, and (2) the Unc5s family, including Unc5A through D. Notably, Netrins can also bind to integrins, a large family of transmembrane heterodimeric receptors that comprise a broad set of functions, among them, joining the actin cytoskeleton to extracellular matrix (ECM) proteins [10]. Thus, Netrins acting on different receptors lead to multifunctional effects through different signaling mechanisms, depending on the developmental stage and/or tissue involved. It has been reported that Netrin-1 requires intracellular signaling mechanism involving RhoA (member of the small GTPase family), Fyn, focal adhesion kinase (FAK), among others [11]. These mechanisms have been shown in the context of cancer progression [12], branching and membrane extension on oligodendrocyte [11], axon outgrowth in neurons [13], and osteoclast differentiation in bone [14]. While several reports demonstrate that Netrin-1 is a key angiogenic promoter, other researchers highlight its ability to negatively regulate capillary branching in the developing vascular system and to reduce endothelial cell migration in vitro [5, 15]. For example in the human placenta, the most vascularized mammalian organ; Netrins have different angiogenic implications according to its localization and distribution pattern in first trimester and term placentas [16, 17].

Angiogenesis plays a key role during embryonic development, but also is involved in postnatal wound-healing processes. When a tissue is damaged, several events, such as cell migration and proliferation, occur to recover tissue integrity. These events involve extracellular matrix deposition, angiogenesis, and tissue remodeling [18]. If the recovering tissue is not properly vascularized, the healing process can be impaired. Therefore, studying the mechanisms that modulate angiogenesis is necessary for the development of therapeutic tools in order to be able to induce proper wound healing in affected patients.

In this context, researchers have demonstrated that multipotent mesenchymal stromal cells (MSCs) not only improve tissue repair in response to injury and disease, but also induce angiogenesis in damaged tissues [19–21]. However, the molecular mechanisms underlying this beneficial effect are not fully understood. It has been reported that MSCs do not differentiate at wound locations; they have low grafting efficiency and rather act by secreting a broad set of growth factors, cytokines, chemokines, and bioactive lipids into the wound microenvironment [22]. This suggests that MSCs have a predominantly paracrine/autocrine signaling effect, and are not particularly involved in lineage differentiation [21, 23, 24]. We have recently demonstrated that MSCs isolated from the stromal tissue of the umbilical cord, named Wharton’s Jelly mesenchymal stem cells (WJ-MSC) present enhanced angiogenic properties when compared to MSCs isolated from adipose tissue (AD-MSC). We showed that WJ-MSC promote angiogenic processes via a paracrine mechanism, by expressing and secreting a broad set of classic angiogenic factors, such as vascular endothelial growth factor (VEGF), platelet-derived growth factor AA (PDGF-AA), transforming growth factor beta 1 (TGF-β2), basic fibroblast growth factor 2 (bFGF), and hepatocyte growth factor (HGF), which are directly and/or indirectly linked with angiogenesis [19]. Our results suggest that WJ-MSC are a primitive stromal cell population, with therapeutic potential, due to the fact that they expanded faster and to a greater extent than adult-derived MSCs. However, the presence and secretion of other non-canonical angiogenic molecules, such as Netrins, has not yet been explored. Moreover, to our knowledge, there is no description to date of how Netrin receptors present in endothelial cells may modulate pro- and/or anti-angiogenic effects of these ligands.

Accordingly, the aim of this work was to elucidate if WJ-MSC-secreted Netrin-1 could exert an angiogenic role both in vitro in primary cultures of human umbilical vein endothelial cells (HUVEC) and in vivo in chicken embryo chorioallantoic membrane (CAM assay). We here show for the first time that Netrin-1, expressed and secreted by WJ-MSC, acts as a non-canonical angiogenic factor. Moreover, we show that integrin α6β1, Unc5b, and Neogenin-1 are the receptors that might participate preferentially in regulating angiogenesis. Overall, our results point to the dynamic balance between the pro- and anti-angiogenic properties of Netrin-1 highlighting the importance of the type and levels of Netrin-1 receptors present locally in the final response. Still, there is a need to further understand the mechanisms of action involved in the observed angiogenic phenomenon. Undoubtedly, these results have therapeutic potential in the development and optimization of strategies aiming to manipulate angiogenic processes for the treatment of a wide number of pathologies.

## Methods

### Isolation of human mesenchymal stem cells from Wharton’s jelly of the umbilical cord (WJ-MSC)

Umbilical cords used for WJ-MSC isolation were provided by Dr. Luis Tisné Brousse Hospital, Santiago, Chile. Healthy pregnant women attending routine antenatal care at the maternity center offered to participate voluntarily in this study. The inclusion criteria for selected women were: non-smokers, normotensive, normal cholesterol levels, not having pre-eclampsia, pregestational nor gestational diabetes mellitus*,* nor a family history of premature vascular diseases, and no regular consumption of medication. Written consent from these patients was obtained. The ethics committee of the University of Chile and Dr. Luis Tisné Brousse Hospital approved this protocol.

Within 24 h umbilical cords were processed in our laboratory following standard procedures [19, 25]. Briefly, the umbilical cord was dissected to discard blood vessels, then was cut into 2-mm^2^ pieces and digested with collagenase I (1 μg/μL, Gibco by Life Technologies, Carlsbad, CA, USA) in phosphate-buffered saline (PBS, pH 7.4) with gentle agitation at 37 °C for 16 h in order to disaggregate the tissue. The cells obtained by subsequent centrifugation (2000 rpm, 10 min) were then washed and seeded in Dulbecco’s modified Eagle’s medium (DMEM) (Life Technologies) containing 10% fetal bovine serum (FBS) (Hyclone, Logan, UT, USA) with antibiotics (100 U/mL penicillin/streptomycin, Thermo Fisher Scientific, Waltham, |MA, USA) and maintained in this condition for 24 h at 37 °C, 5% CO_2_. Afterwards, non-adherent cells were discarded and adherent cells were incubated at 37 °C, 5% CO_2_, changing the medium every 2–3 days. All primary cultures of WJ-MSC were used between passages 2–5. Human adipose tissue-derived mesenchymal stem cells (AD-MSC) and human bone marrow-derived mesenchymal stem cells (BM-MSC), kindly donated by Dr. Montencinos, were cultivated in the same conditions as WJ-MSC.

### Human umbilical vein endothelial cells (HUVEC) isolation and culture

HUVEC were obtained from full-term normal umbilical cords as described [26]. Briefly, umbilical veins were rinsed with warm (37 °C) phosphate-buffered saline solution (PBS, in mM: NaCl 136, KCl 2.7, Na_2_HPO_4_ 7.8, KH_2_PO_4_ 1.5, pH 7.4) and endothelial cells were isolated by collagenase (0.2 mg/mL) digestion and cultured (37 °C, 5% CO_2_) up to passage 2 in medium 199 (M199) supplemented with 10% newborn calf serum, 10% fetal calf serum, 3.2 mM L-glutamine and 100 U/mL penicillin-streptomycin. The medium was changed every 2 days until confluence was reached. All primary cultures of HUVEC were used between passages 2–5.

### Conditioned media precipitation and Netrin-1 determination

In order to evaluate the secretion of Netrins by WJ-MSC, conditioned media were collected after 48 h of culture in serum starvation. To analyze the samples, through Western blotting, we concentrated the proteins secreted by the cultured cells. Briefly, conditioned media was distributed in aliquots of 1 mL. Next, 500 μL of methanol at −20 °C was added and vortexed for 30 s, then 125 μL of chloroform was added following a final vortex step of 20 s, medium was centrifuged at 14,000 rpm for 5 min. Finally, the interface was recovered, and suspended in 25 μL of loading buffer. The pellets were frozen at −20 °C until further use. The same sample of conditioned media was used to evaluate Netrin-1 levels secreted by WJ-MSC through ELISA (USCN Life Science Inc., Houston, TX, USA).

### Histological analysis and immunohistochemistry

Umbilical cords were recovered and fixed with 4% paraformaldehyde for 8 h, dehydrated with graded alcohols (75–100%) and paraffin embedded. Next, 12-μm sections underwent treatment with proteinase K for antigen retrieval and were then treated with 3% hydrogen peroxide in methanol to block endogenous peroxidase activity. After washing twice with PBS, sections were incubated with horse serum for 1 h and then incubated for 1 h at 37 °C with anti-Netrin-1 or −4 (R&D Systems, Minneapolis, MN, USA). Next, sections were washed twice with PBS before incubation with the secondary antibody for 20 min at 37 °C. Then, the sections were incubated with ABC solution (Vectastain ABC Kit, Vector Laboratories, Burlingame, CA, USA) for 20 min at 37 °C, washed twice with PBS and the reaction developed using DAB (Vector Laboratories). Sections were stained in hematoxylin (Vector Laboratories) and eosin Y (Sigma-Aldrich, St. Louis, MO, USA) and mounted.

### Flow cytometry analysis

WJ-MSC cultures from different donors were analyzed by flow cytometry (FACSCanto II, BD Biosciences, San Jose, CA, USA) in order to evaluate the presence of classical MSC markers using same conditions as previously described [19]. In addition, we analyzed for Netrin-1 and Netrin-4 and the CD29 non-classical receptor.

### Confocal microscopy

WJ-MSC or HUVEC monolayers were grown on Lab-Tek® chamber slides with cover (Nunc, Naperville, IL, USA) up to 80% confluence, then rinsed in Hanks’ solution and fixed in 4% paraformaldehyde (15 min). Fixed cells were rinsed with Hanks’ solution, permeabilized with 0.1% Triton X-100 (20 min), and blocked (1 h) with 1% bovine serum albumin (BSA). Monolayers were incubated for 30 min at 37 °C with combinations of the following antibodies: Netrin-1 and −4 (R&D Systems), Neogenin-1 and eNOS (Santa Cruz Biotechnology, Dallas, TX, USA), UNC5b (Cell Signaling, Danvers, MA, USA), CD29 (Thermo Fisher Scientific), VEGF (Abcam, Cambridge, MA, USA) or CD31 (Sigma-Aldrich) followed by incubation with the corresponding secondary antibodies (Alexa Fluor 488 and 555; Molecular Probes, Eugene, OR, USA). Nuclei were stained with 4’, 6-diamidino-2-phenylindole (DAPI) (Sigma-Aldrich). Fluorescent secondary antibodies were visualized with Zeiss LSM 510 META (Carl Zeiss Microscopy GmbH., Jena, Germany).

### RNA isolation and real-time polymerase chain reaction (qPCR)

Total RNA was isolated using the Thermo Fisher Scientific kit following manufacturer’s instructions. RNA quality and integrity were insured by gel visualization and spectrophotometric analysis (OD_260/280_), quantified at 260 nm. Aliquots of 1 μg of total RNA were retro-transcribed into cDNA using dNTPs (10 mM) plus Random Primer (3 μg/μL) and RevertAid™ Reverse Transcriptase 200 U/μL. qPCR experiments were performed using 1 ng of cDNA as template, SYBR Green Mix and a Stratagene Mx3000P real-time PCR system (Agilent Technologies, Santa Clara, CA, USA). Primers were designed for specific amplification of different receptors and ligands (Additional file 1: Table S1). HotStart was activated (15 min, 95 °C) and assays included a 95 °C denaturation (15 s), annealing (20 s) at 60 °C, and extension at 72 °C (10 s). Fluorescent products were detected after an additional 3-second step to 5 °C below the product melting temperature (*T*_m_). Product specificity was confirmed by agarose gel electrophoresis (2% v/v) and melting curve analysis. Amplification analysis was carried out with the MxPro software (Stratagene, San Diego, CA, USA) and relative quantification was calculated using the ∆∆CT method. *GAPDH* was used as a housekeeping gene, former confirmation that its expression levels did not change between experiments.

### Tube formation assay

The tube formation assay was used as a model for assessment of endothelial migration, a critical parameter in angiogenesis. Matrigel (BD Matrigel; BD Biosciences) was thawed overnight at 4 °C and administered by cold tips in 50 μL per well in cold 96-well cell culture plates. Matrigel made a thin gel layer after incubation at 37 °C for 1 h. A total of 55,000 cells were seeded on each Matrigel-coated well, and after 4 h network formation was evaluated under a light microscope. Results represent experiments carried out in duplicate at least three times. Matrigel experimental conditions included CBO (a VEGF-receptor inhibitor, 20 μM, Calbiochem, San Diego, CA, USA), IgG (an isotype antibody control, 2 μg/mL), 2F5 (a novel drug targeting Netrin-1, 2 μg/mL, kindly provided by Dr. Mehlen), anti-Netrin-1 antibody (R&D Systems, 2 μg/mL) and exoenzyme C3 transferase (RhoA inhibitor, Cytoskeleton, Inc., Denver, CO, USA, CT04 1–1.5 μg/mL), in absence or presence of recombinant human Netrin-1 (R&D Systems, 10 pg/mL–1000 ng/mL); all in endothelial basal media (EBM). In order to inhibit basal secretion of VEGF and Netrin-1, HUVEC were cultured 1 h with CBO, 2F5 or exoenzyme C3 transferase prior to each experiment and once after Matrigel polymerization.

### Wound-healing assay

HUVEC were used to study the paracrine effect of WJ-MSC-conditioned media (48 h) and recombinant human Netrin-1 (10–1000 ng/mL) on endothelial cell migration potential through a wound-healing assay. Immediately after scratching the confluent HUVEC monolayer using 200-μL sterile tips, wound healing was initiated by administering recombinant human Netrin-1 (R&D Systems) in different wells of a 24-well plate. Photographs were taken 8 and 24 h after wounding and area of remaining scratched zone was measured.

### Western blot

Primary cell cultures of HUVEC or WJ-MSC were harvested in monolayers or seeded in Integra® Matrix scaffold (IM; Integra® LifeSciences Corp., Plainsbro, NJ, USA) or a home-made collagen I (Col) solution obtained from Sprague-Dawley rat tails, as previously described [19]. Homogenates were suspended in SDS lysis buffer with added protease and phosphatase inhibitors at 4 °C. Equal amounts of protein were separated by 8–12% SDS-PAGE, followed by Western blotting using anti-Netrin-1 (66 kDa) and −4 (70–75 kDa) (R&D Systems), anti-VEGF (43–45 kDa) and anti-UNC5c (103 kDa) (Abcam), anti-DCC (180–190 kDa) and anti-phospho-ERM (index of activated RhoA/ROCK signaling) (75–80 kDa) (BD Transduction Laboratories), anti-UNC5b (130 kDa) (Cell Signaling), anti-Neogenin-1 (175 kDa) and anti-RhoA (24 kDa) (Santa Cruz Biotechnology). The monoclonal mouse anti-β actin (42 kDa) (Sigma-Aldrich) antibody was used as loading control. Membranes were washed in Tris-buffered saline (TBS) with 0.1% Tween, and incubated (1 h, 22 °C) in TBS/0.1% Tween containing horseradish peroxidase-conjugated goat anti-rabbit, anti-sheep or anti-mouse secondary antibodies. Bands were visualized using enhanced chemiluminescence (ECL; Amersham Biosciences, Little Chalfont, UK).

### Chicken chorioallantoic membrane (CAM) assay

WJ-MSC (1.5*10^6^) were used in CAM assays. The experimental approaches included: DMEM (20 μL), 2F5 (0.5 μg/μL, kindly provided by Dr. Mehlen), CBO (50 μM, Calbiochem), C3 transferase (Cytoskeleton, Inc., CT04 1–1.5 μg/mL) and recombinant human Netrin-1 (R&D Systems, 10 ng/mL). Fertilized chicken eggs (Rock iso, Agricola Chorombo, Santiago, Chile) were incubated at 38.5 °C with 75% RH. At embryonic day 3 (E3), eggs were wiped with 70% ethanol and 3 mL of albumin were extracted from each egg, after which eggs were returned to the incubator. On E4, a window of 2 cm^2^ was opened and pen/strep (250 μL, 100 U, 100 mg; Invitrogen) was applied prior to returning to the incubator. On E7, a plastic ring (6 mm in diameter and 2 mm in height) was inserted and on E8, WJ-MSC (1.5*10^6^) were placed over the CAM, after which an initial image was obtained. Angiogenic response was imaged every 24 h with a digital camera HD IC80 (Leica, Wetzlar, Germany). DMEM was used as negative control. We used the perimeter inside of the plastic ring, where the stimulus was applied in the study area. In order to quantify the number of branches of the blood vessels crossing this area, we used the software Image J (NIH, Bethesda, MD, USA). In E12, cream was added below the CAM before photographing as previously described [19].

### Statistical analysis

All the determinations were carried out in triplicate. Values are mean ± S.E.M., where n indicates number of independent cell cultures isolated from different umbilical cords (n = 3–7). Comparisons between two and more groups were performed by means of Student’s unpaired *t* test and analysis of variance (ANOVA), respectively. If the ANOVA demonstrated a significant interaction between variables, post hoc analyses were performed by multiple-comparison Bonferroni correction test. The software Graphpad Prism 5.0b (GraphPad Software Inc., San Diego, CA, USA) was used for data analysis. *p* < 0.05 was considered statistically significant.

## Results

### WJ-MSC express and secrete non-classical angiogenic factors

**Fig. 1:**
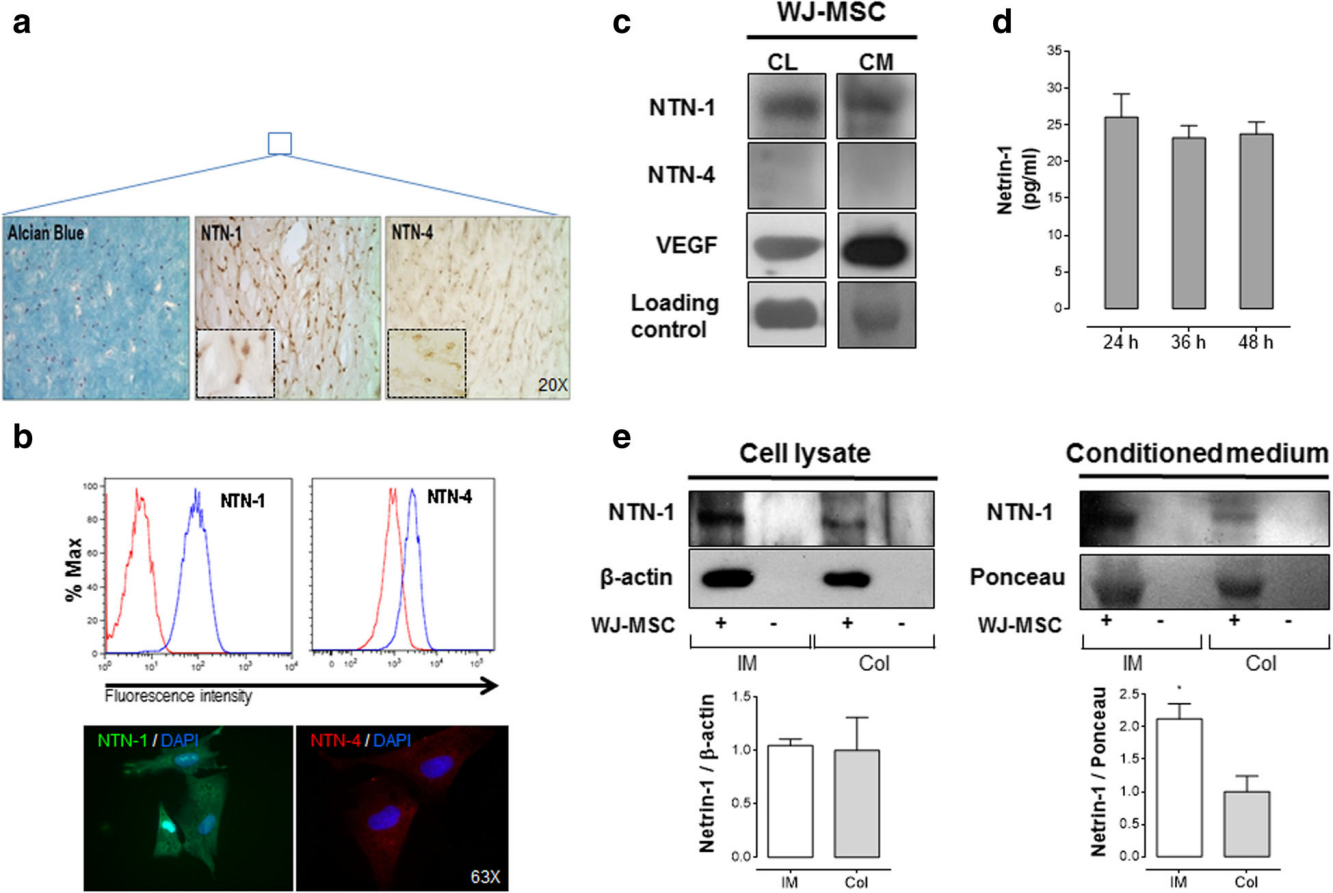
Netrin-1 is preferentially expressed and secreted by Wharton’s jelly-derived mesenchymal stem cells (WJ-MSC), whilst Netrin-4 expression is almost absent. Netrin-1 (NTN-1) and Netrin-4 (NTN-4) expression was confirmed by different experimental techniques. **a** Representative images of the histological analysis of umbilical cord sections are shown. MSCs uniformly distribute within Wharton’s jelly and show positive staining for Alcian Blue and Netrins (magnification × 20). Inset show optical zoom images of NTN-1 and NTN-4 staining (*n* = 3). **b** Flow cytometry and immunofluorescence anti-NTN-1 and −4 confirm preferential expression of NTN-1 in WJ-MSC (*n* = 3) (magnification × 63). **c** Western blot for NTN-1, NTN-4 and vascular endothelial growth factor (VEGF) (positive control) in whole cell lysate (CL) and conditioned media (CM) of WJ-MSC cultures. β-actin (for CL) and Ponceau (for CM) were used as internal loading controls, respectively (*n* = 3–7). **d** ELISA analysis for NTN-1 in WJ-MSC; CM was obtained at indicated time points (*n* = 4). **e** Western blot for NTN-1 in WJ-MSC, seeded 24 h in Integra® matrix (IM) or collagen I (Col), versus respective empty scaffold (−) used as negative control. Ligand expression was analyzed both in CL or CM. β-actin and Ponceau were used as internal loading controls, respectively (*n* = 4–5)

The expression of Netrins was first characterized in Wharton’s jelly umbilical cord sections (Fig. 1a). We detected preferentially the presence of Netrin-1 but not of Netrin-4. These results were corroborated by qPCR experiments (Additional file 1: Figure S1A) and confirmed by flow cytometry and immunofluorescence on WJ-MSC cultures (Fig. 1b). Netrin-1 is highly expressed throughout the early passages (Additional file 1: Figure S1B) and can also be detected in AD-MSC and BM-MSC (Additional file 1: Figure S1C). Furthermore, both intracellular Netrin-1 production and secretion into conditioned media could be detected, similar to VEGF, used as a positive control. Nevertheless Netrin-4 was not detected (Fig. 1c). We found that Netrin-1 secretion by WJ-MSC measured by ELISA assay was in the 25–30 pg/mL range (Fig. 1d), similar to recently reported levels in the umbilical cord blood [27]. In order to evaluate if WJ-MSC produce Netrin-1 when confined in a three-dimensional matrix more similar to their in vivo niche, cells were seeded in two types of scaffolds: Integra® matrix (IM) or collagen I (Col). We found that Netrin-1 synthesis after 24 h in both matrices was equally detected in cell lysate. However, the Netrin-1 secretion levels in the conditioned media were higher when cells were seeded in IM compared to Col (Fig. 1e). This is consistent with our previous results showing that WJ-MSC improve their angiogenic capabilities on IM [19].

### Netrin-1 promotes endothelial cell migration and pro-angiogenic response

**Fig. 2:**
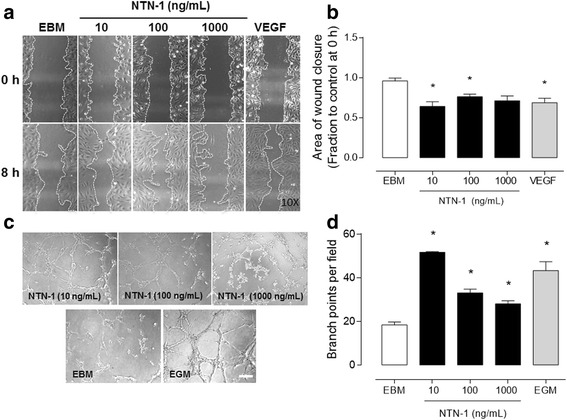
Netrin-1 induces angiogenesis in vitro in HUVEC. **a** Netrin-1 (NTN-1) influence on HUVEC cell migration was determined using scratch assay, where cells were serum-starved and treated for 8 h with different recombinant human NTN-1 concentrations, as indicated. Endothelial basal media (EBM) was used as internal reference and recombinant human vascular endothelial growth factor (VEGF) (40 ng/mL) was used as a positive control. Representative pictures of each condition are shown (amplification × 10). **b** Quantified results correspond to the mean ± S.E.M. (*n* = 3, ^*^ *p* < 0.05). **c** The effect of recombinant human NTN-1 was determined in vitro to evaluate angiogenesis through tubule formation assay. HUVEC were serum-starved and seeded on Matrigel and treated with increased concentrations of NTN-1 for 4 h. EBM alone was used as internal reference and endothelial growth media (EGM) was used as a positive control. Representative images are shown for each experimental condition (scale bar = 15 μm). **d** Quantified results correspond to the mean ± S.E.M. (*n* = 6, ^*^ *p* < 0.05)

Having characterized the expression of canonical and non-canonical angiogenic factors in WJ-MSC, we next decided to study the ability of recombinant human Netrin-1 to induce a pro-angiogenic response in primary endothelial cells [17]. In particular, we evaluated cell migration in response to different Netrin-1 concentrations using the wound-healing assay on HUVEC. As shown in Fig. 2a, the wound area at 8 h decreased only at the lowest ligand concentrations, i.e., 10 and 100 ng/mL of Netrin-1, corresponding to the reported physiological concentration range [3, 28]. It is noteworthy to mention that Netrin-1 effect on HUVEC migration was similar to VEGF, 59.6% vs. 60%, respectively (Fig. 2b). Indeed the WJ-MSC secretome had pro-angiogenic potential as verified by a wound-healing assay being equal to the positive control VEGF in increasing the closure of a wound in primary HUVEC cultures (Additional file 1: Figure S2). As an alternative method to study the angiogenic properties of recombinant human Netrin-1, we used the two-dimensional tubule formation assay in which endothelial cells organize into tubular structures in response to an angiogenic stimulus. We found that Netrin-1 promoted tube formation in a dose-dependent manner, with low concentrations (10 ng/mL) having a greater effect than higher doses (100–1000 ng/mL). The latter indicates that at high doses Netrin-1 could lose its ability of inducing endothelial cell migration and even prevent endothelial cell movements (Fig. 2c–d). These results are consistent with other studies that suggested a pro-angiogenic role for Netrin-1 at physiological concentrations [3, 29, 30]. Hence, we studied the angiogenic properties of lower dose-response curves of recombinant human Netrin-1. Notably, the tubule formation and branch points were promoted in a dose-dependent manner even in the pg/mL range (Additional file 1: Figure S3).

### Endogenous Netrin-1, present in WJ-MSC-conditioned medium, promotes tubule formation in endothelial cells

**Fig. 3:**
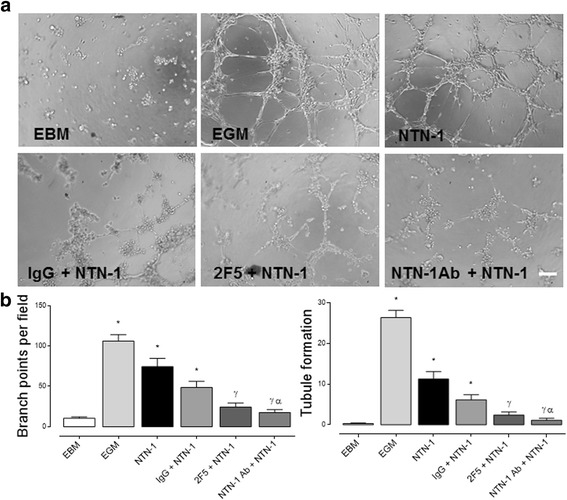
Netrin-1 promotes angiogenesis in HUVEC. **a** Representative images of HUVEC tubule assay. Cells were exposed for 4 h to endothelial basal media (EBM), endothelial growth media (EGM), IgG (internal antibody control, 2 μg/mL), 2F5 [a drug targeting Netrin-1 (NTN-1), 2 μg/mL] and anti-NTN-1 antibody (R&D Systems, 2 μg/mL), in absence or presence of recombinant human NTN-1 (10 ng/mL), scale bar = 15 μm. **b** Quantified data correspond to the mean ± S.E.M. (*n* = 4, ^*^ *p* < 0.05 vs. EBM, ^γ^ *p* < 0.05 vs. NTN-1, ^α^ *p* < 0.05 vs. IgG + NTN-1)

**Fig. 4:**
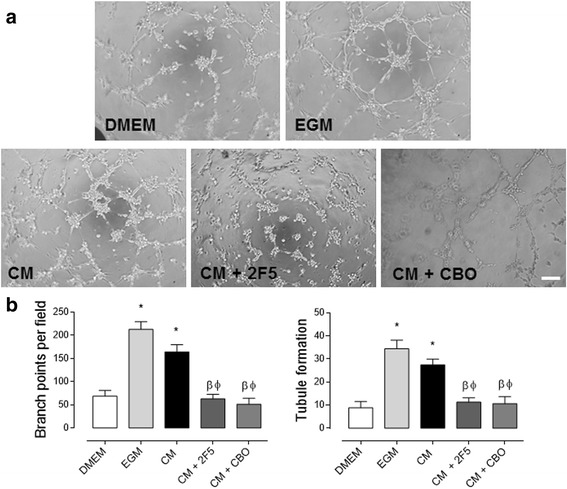
Netrin-1, secreted by WJ-MSC, promotes angiogenesis in HUVEC. **a** Representative images of HUVEC tubule assay treated as indicated. Cells were exposed for 4 h to DMEM, endothelial growth media (EGM), WJ-MSC-conditioned media (CM), 2F5 [a drug targeting Netrin-1 (NTN-1), 0.5 μg/μL] and CBO (a VEGF-receptor inhibitor, 20 μM), scale bar = 15 μm. **b** Quantified data correspond to the mean ± S.E.M. (*n* = 3, ^*^ *p* < 0.05 vs. DMEM, ^β^ *p* < 0.05 vs. EGM, ^Φ^ *p* < 0.05 vs. CM)

WJ-MSC-conditioned media proved to be angiogenic, corroborating our former experiments [19]. We tested the contribution of Netrin-1 present in the WJ-MSC-conditioned media (48 h) by comparing the effect to recombinant human Netrin-1 (10 ng/mL), concentration that induced the maximal physiological angiogenic response, to the one elicited by VEGF. Consistent with VEGF being an important mediator of this response, the addition of a VEGF-receptor inhibitor (CBO, 20 μM) [31] decreased its pro-angiogenic effect, as observed even with VEGF co-treatment (40 ng/mL) (Additional file 1: Figure S4). Next we treated WJ-MSC with VEGF-receptor inhibitor (CBO, 20 μM), a drug targeting Netrin-1 (2F5, 2 μg/mL) [32], a neutralizing Netrin-1 antibody (R&D Systems, 2 μg/mL) [33] and isotype control antibody (IgG, 2 μg/mL). We found that recombinant human Netrin-1 and IgG, as well as the internal positive control-endothelial growth medium (EGM) promoted an angiogenic effect on HUVEC when compared to endothelial basal medium (EBM). On the other hand, both 2F5 and neutralizing Netrin-1 antibody inhibited significantly the pro-angiogenic responses on HUVEC (Fig. 3a–b). Likewise, WJ-MSC-conditioned media (48 h) and recombinant human Netrin-1 (10 ng/mL) increased tubule formation and branch points. Nonetheless, 2F5 and CBO inhibited these pro-angiogenic responses in each condition on HUVEC (Fig. 4a–b). Of note, when excluding the VEGF contribution with the use of the CBO inhibitor, we still could detect a pro-angiogenic effect on HUVEC with WJ-MSC-conditioned media, suggesting an important contribution of non-canonical pro-angiogenic factors such as Netrin-1.

### Netrin-1 induces angiogenesis in vivo in a CAM assay

**Fig. 5:**
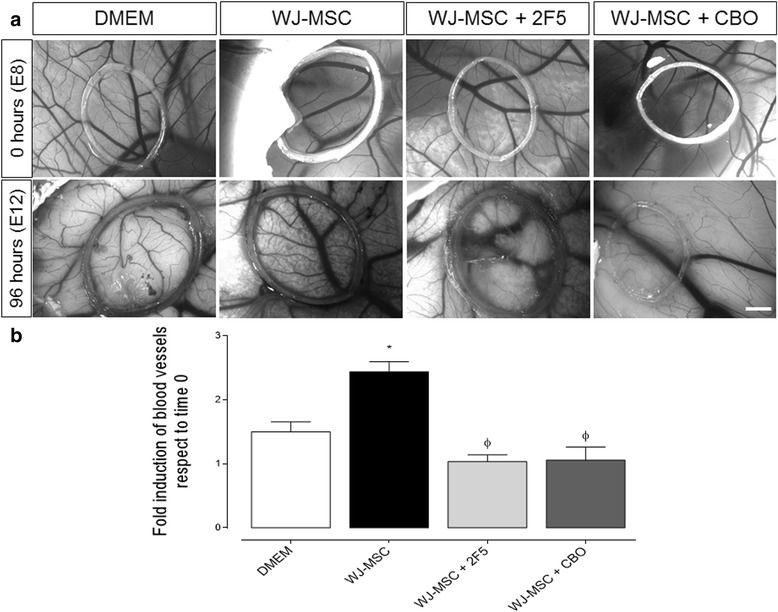
Netrin-1 contributes to angiogenesis in vivo in a CAM assay. **a** Representative images of distinct experimental approaches on CAM: DMEM (20 μL), Wharton’s jelly-derived mesenchymal stem cells (WJ-MSC) (1.5*10^6^), 2F5 [a drug targeting Netrin-1 (NTN-1), 0.5 μg/μL] and CBO (a VEGF-receptor inhibitor, 50 μM), using for each condition approximately 15 eggs (scale bar = 1 mm). **b** Quantification of angiogenesis after 4 days of incubation in experimental conditions as indicated. Data correspond to the mean ± S.E.M. (WJ-MSC, *n* = 5, ^*^ *p* < 0.05 vs. DMEM, ^Φ^ *p* < 0.05 vs. WJ-MSC)

Having evaluated the in vitro effect that Netrin-1 secreted by WJ-MSC exerts, we extended our observations by assessing its contribution to the angiogenic response on a chicken chorioallantoic membrane (CAM) assay in vivo (Fig. 5a). We found that WJ-MSC induced functional blood vessel formation compared to control (DMEM), an effect that remained after 96 h of stimulus. A different effect was observed when a Netrin-1-targeting drug (2F5, 0.5 μg/μl) [32], or VEGF-receptor inhibitor (CBO, 50 μM) [34] were placed on WJ-MSC confined in the plastic ring, because both drugs decreased the blood vessel formation by approximately 50% (Fig. 5b). These results indicate that WJ-MSC synthesize pro-angiogenic factors including not only canonical ones [19] but also non-canonical such as Netrin-1, fostering the growth of newly recruited endothelial cells from the CAM.

### Endothelial cells of the umbilical vein express low amounts of the Netrin’s classical receptors Neogenin-1, UNC5b and UNC5c, but not DCC

**Fig. 6:**
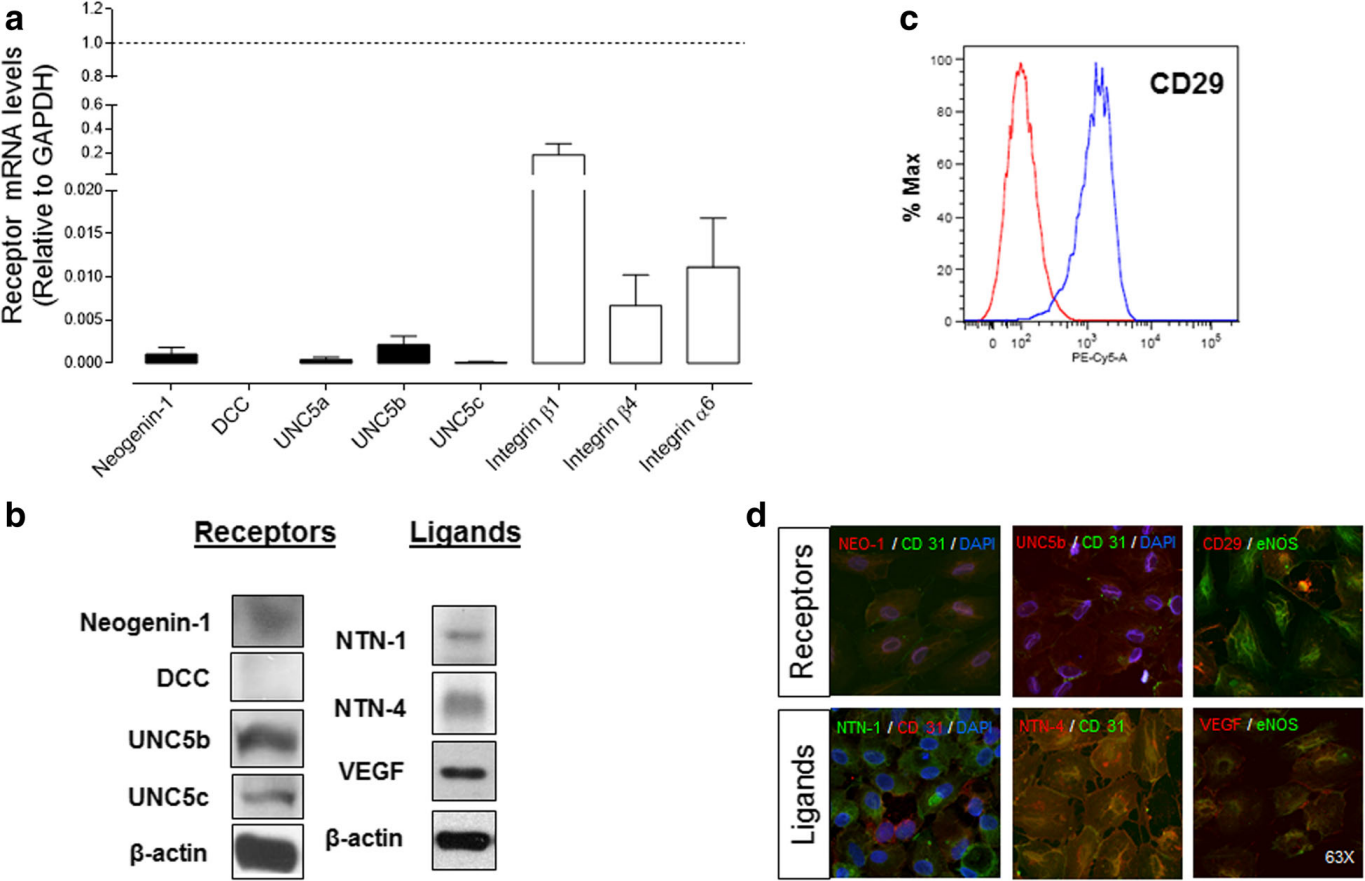
Endothelial cells derived from human umbilical vein endothelial cells (HUVEC) express low levels of classic Netrin receptors and both Netrin-1 (NTN-1) and Netrin-4 (NTN-4) ligands. **a** mRNA levels of classic (■) and non-classic (□) Netrin receptors were quantified by qPCR relative to *GAPDH* expression (*dashed line*). Values are mean ± S.E.M. (*n* = 7). **b** Absence of DCC expression in HUVEC was established by Western blot, while Neogenin-1 as well as UNC5b and UNC5c expression could be detected. Endogenous Netrins and vascular endothelial growth factor (VEGF) expression in HUVEC was determined by Western blot. β-actin was used as internal reference (*n* = 5–7). **c** The non-classical Netrin receptor integrin α3β1 was also detected by flow cytometry (*n* = 3). **d** Immunofluorescence for receptors and ligands in HUVEC (magnification × 63). eNOS (endothelial nitric oxide synthase) or CD-31 (PECAM) were used as endothelial cell markers and DAPI for nuclear counterstain

Even though Netrin-1 has been widely studied in endothelial cells, discrepancies exist about which of the receptors that bind Netrin-1 could be involved in the angiogenic processes. Because HUVEC responded to Netrin-1, we explored the possible candidate of classical and non-classical receptors. In addition, we also explored the expression of Netrins by HUVEC itself (Fig. 6a–d). We evaluated by qPCR expression of *Neogenin-1, DCC, UNC5 a, b, c and integrins β1, β4, α6*. Neogenin-1, DCC, UNC5b, and UNC5c protein expression was also assayed by Western blot. Of note, among the classical receptors only Neogenin-1, UNC5b and UNC5c were detectable in HUVEC. Furthermore, in line with its recently described Netrin receptor function, integrin expression was also found, and further confirmed by flow cytometry in HUVEC [8, 35]. Moreover, the endogenous Netrins contribution in HUVEC, as assayed by Western blot showed that these cells express actually more Netrin-4 than Netrin-1, but both expression levels remain lower than that of VEGF (Fig. 6b, d). In fact, HUVEC-conditioned medium (48 h) did not have any contribution in the angiogenic response observed in vitro, ruling out an autocrine contribution (data not shown).

### Netrin-1 contribution to angiogenesis is not mediated by endothelial RhoA/ROCK signaling

**Fig. 7:**
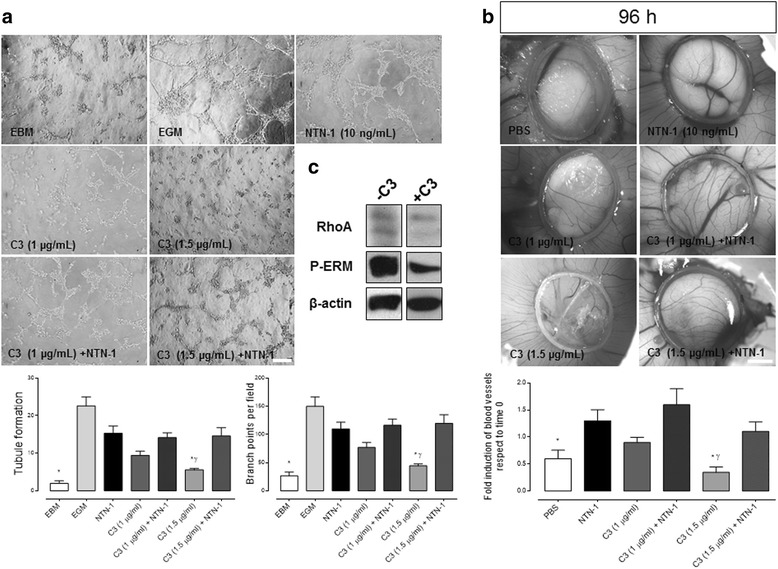
RhoA/ROCK pathway contributes to angiogenesis in vitro and in vivo in a Netrin-1-independent manner. **a** Representative images of HUVEC tubule assay. Cells were exposed for 4 h to endothelial basal media (EBM), endothelial growth media (EGM) and exoenzyme C3 transferase (Cytoskeleton, Inc., 1–1.5 μg/mL), in absence or presence of recombinant human Netrin-1 (NTN-1) (10 ng/mL). The graphs shown below represent quantified data corresponding to the mean ± S.E.M. [*n* = 3, ^*^ *p* < 0.05 vs. NTN-1, ^γ^ *p* < 0.05 vs. C3 + NTN-1], scale bar = 15 μm. **b** Representative images of distinct experimental approaches on CAM: PBS (20 μL), NTN-1, 10 ng/mL) and exoenzyme C3 transferase (Cytoskeleton, Inc., 1–1.5 μg/mL) (scale bar = 1 mm). The graph represents quantification of angiogenesis after 4 days of incubation in experimental conditions as indicated. Data correspond to the mean ± S.E.M. (*n* = 4, ^*^ *p* < 0.05 vs. NTN-1, ^γ^p < 0.05 vs. C3 + NTN-1). **c** Western blot for RhoA, phospho-ezrin-radixin-moesin (P-ERM, index of activated RhoA/ROCK signaling) and β-actin in primary culture of HUVEC (n = 1) in absence (−) or presence (+) of exoenzyme C3 transferase (Cytoskeleton, Inc., 1.5 μg/mL)

Netrin-1 and its receptors could regulate angiogenesis by activating a variety of intracellular pathways [12]. Neogenin-1 and Unc5b are both moderately expressed in HUVEC. Both receptors signal through RhoA/ROCK pathway activation [13]. Thus, in order to gain insight into the molecular mechanism underlying Netrin-1 function in HUVEC, we evaluated if the RhoA/ROCK pathway was involved in the angiogenic responses observed in HUVEC. By using a highly potent reagent that targets endogenous RhoA protein, exoenzyme C3 transferase, we evaluated the contribution of this signaling cascade in Netrin-1-driven angiogenesis. We found that both in vitro (Fig. 7a) and in vivo (Fig. 7b) RhoA/ROCK modulates angiogenesis, since pharmacological inhibition of the pathway using 1.5 μg/mL of exoenzyme C3 transferase disturbs tubule formation significantly. As expected, this lower RhoA/ROCK activity was in accordance with a decrease in ERM phosphorylation (Fig. 7c). Yet using Netrin-1 in combination with exoenzyme C3 transferase did not diminish the Netrin-1 angiogenic response, revealing that probably other downstream signaling effectors are involved (Fig. 7a–b).

## Discussion

Stem cell-based therapy plays an important role in the treatment of various diseases. Many authors have shown that MSCs have a significant effect on angiogenesis [36]. We recently demonstrated that WJ-MSC seeded on bioartificial scaffolds could improve wound healing in vitro and in vivo [19].

Previous studies have explored the possible role in embryonic vasculogenesis and angiogenesis of a new group of molecules. The Netrin protein family, which acts in the nervous system in axonal guidance [3], has later been found to be implicated in angiogenesis [37], cell survival [38], and morphogenesis [39]. Moreover, recent studies suggest that Netrin-1 may become a therapeutic target in the treatment of various pathologies, either as a stimulant or inhibitor of angiogenesis such as in diabetes or cancer, respectively, even protecting the heart against ischemia-reperfusion injury [2, 40, 41]. However, the underlying cellular mechanisms and receptor specificity characteristics involved in angiogenesis still require further studies.

In the present study, we show for the first time that Netrin-1 is produced and secreted by WJ-MSC throughout different passages, and has the ability to stimulate cell migration and motility in endothelial cells. We present different approaches for the detection of the non-canonical angiogenic factor, Netrin-1 in WJ-MSC. Using conditioned medium, as well as cell homogenates from WJ-MSC cultures, our data show that this type of cell is able to express and secrete Netrin-1 while Netrin-4 is almost undetectable. Interestingly, *Netrin-4* mRNA is revealed in other MSC populations, presenting the highest expression in BM-MSC [42]. Notably, our Western blot analysis revealed that all assayed MSC sources express Netrin-1 protein. So far, no evidence exists indicating Netrin-1 expression in AD-MSC or BM-MSC. However, Netrin-1, acting through its receptor UNC5b, has been involved in osteoclast differentiation in the bone marrow [43]. On the other hand, in obese adipose tissue, the ligand recently has been implicated in reduced migratory capacity of macrophages, increasing its accumulation in adipose tissue [44]. Moreover, Netrin-1 was informed to be highly expressed in obese but not lean adipose tissue of humans and mice, acting as a neuroimmune guidance cue via its receptor UNC5b. The latter could explain our observation showing a lower expression of Netrin-1 in AD-MSC in comparison to BM-MSC. Nevertheless, at this point we cannot infer that Netrin-1 is differentially expressed among AD-MSC versus BM-MSC. Recently, in rodents, transplantation of either BM-MSC or AD-MSC with Netrin-1, has shown to improve the function of the sciatic nerve after injury [45] and also to exert a protective role after myocardial infarction [46]. Given the reported beneficial impact of Netrin-1 in tissue repair, the study of Netrin-1 and its cognate receptors in these MSC populations warrant further investigation.

Different researchers have studied the role of Netrin-1 in angiogenesis. Some of them demonstrating that it has a positive, while others, showing a negative angiogenic effect [5, 37]. Revealing its function is complex due to the fact that the ligands are highly dependent on the cellular context, the receptor through which they exert their function, and their relative concentrations. We therefore evaluated the effect of Netrin-1 on angiogenesis on HUVEC primary cultures only after confirming its expression and secretion by WJ-MSC. We found that Netrin-1 promoted migration and angiogenesis in vitro in a dose-dependent manner, peaking around 50 ng/mL. Moreover, Netrin-1 maintained its positive angiogenic effect even when its dose was a thousand times lower (pg/mL) than reported in many experimental setups. These results agree with recent research that highlights the pro-angiogenic role of Netrin-1 at low doses (10–100 ng/mL) [3, 17, 29]. Our data are also in line with research reporting an inhibitory effect of this ligand at high doses (1 μg/mL) in angiogenesis, thus confirming the potential dual role of Netrin-1 [15]. Some of the reported inhibitory effects, although, were observed in cells that are supplied either from single donors or from pooled donor lines (commercially available HUVEC cell lines) incubated in the presence of complete medium, without considering that Netrin-1 is already present within the serum (data not shown, VP personal communication). It is therefore worth mentioning that our study used exclusively HUVEC primary cultures from single donors in serum-starved conditions.

Our results show that WJ-MSC produce and secrete low amounts of Netrin-1 (25–30 pg/mL), similar to those recently established in the umbilical cord blood [27]. We found that the optimum pro-angiogenic effect was between 10–100 ng/mL of recombinant Netrin-1, which is within the admitted physiological range reported to be between 50–150 ng/mL [5]. Our result is therefore consistent with the hypothesis that Netrin-1 stimulates endothelial cell migration in vitro at low concentrations while conversely, at higher concentrations tends to promote endothelial cell migration to a lesser extent. This dose-dependency is termed a biphasic “bell-shaped” dose-response curve [5, 37]. Clearly, to reveal this bifunctional mode of action of Netrin-1 a more comprehensive in vivo investigation is required.

We were also interested in evaluating Netrin-1’s specific contribution to angiogenesis by inhibiting its function. Seeding HUVEC on Matrigel in presence of a drug targeting Netrin-1, 2F5, or antibody-mediated blockade of Netrin-1 [32, 33] suggests that Netrin-1 signaling pathway directs approximately 70% of the angiogenic process. These results are in accordance with other reports that show the same effect using other experimental approaches [7, 47, 48]. We can therefore irrefutably conclude that this ligand is involved in endothelial angiogenesis [7, 17].

Netrin-1 and its numerous receptors regulate different cellular responses by activating a variety of intracellular pathways. In a first attempt to elucidate the underlying signaling mechanisms we focused on Netrin receptor expression in HUVEC. Examining the Netrin’s classical and non-classical receptor repertoire, we found that HUVEC expressed low amounts of Neogenin-1. HUVEC did not express the Neogenin-1 homologue, DCC. The absence of DCC expression in HUVEC has been well documented in the literature [3, 17, 49]. However, much controversy exists regarding the expression of Neogenin-1 in HUVEC. Many studies investigated Neogenin-1 only measuring mRNA levels and protein expression compared to other tissues, mainly to brain tissue that reveals much higher Neogenin-1 levels. Based on these comparisons, researchers conclude that Neogenin-1 is almost undetectable or not present in HUVEC [40, 49]. Nevertherless, our ongoing research suggests that the expression of Neogenin-1 could be of functional relevance (VP, personal communication). Our present findings demonstrate for the first time in primary cell culture of HUVEC that members of the UNC5 family, described as dependence receptors for Netrin-1 [38] are expressed. UNC5 receptors facilitate repulsion and therefore mediate the anti-angiogenic effect in endothelial cells [15, 37]. Nevertheless, recent studies have proposed that uncharacterized receptors located in endothelium have an opposite role to UNC5b in cell migration, which is dependent on dose/concentration of Netrin-1. The later leaves open the possibility that during a disease progression, levels of Netrin-1 might be modulated determining the severity of the disease [2].

We also found expression of integrin α6β1, a member of a large family of transmembrane proteins, which could be considered a non-classical receptor in HUVEC. However, this type of integrin has been defined as Netrin-4 receptor in lymphatic endothelium [50]. Due to the fact that HUVEC express Netrin-4, it is possible that α6βl may interact with this ligand in the fetal endothelium and, depending on dosage, play an anti-angiogenic role. Considering that umbilical cord remains in continuity with placenta, this idea is feasible since recently a group described that Netrin-4 inhibits cell proliferation and angiogenesis in placenta [51].

Following the downstream pathway of Netrin-1, we evaluated the RhoA/ROCK contribution to Netrin-1 receptor activation in endothelial cells. Several reports have implicated RhoA/ROCK downstream signaling in response to Netrin-1 in neuronal migration [13]. Yet here we demonstrate that Netrin-1 can exert control of angiogenesis in an independent manner of RhoA/ROCK. Of note, we cannot rule out that at higher concentrations of exoenzyme C3 transferase, Netrin-1 might command through Rho/ROCK signaling. In fact, the later could be of importance given that Rho is not required for capillary tube formation [52]. However, in this study they used a C3 transferase recombinant (3 μg/mL) for at least 18 h on commercially available HUVEC cell lines. It is noteworthy that we cannot discard Netrin-1 downstream signaling with other proteins including Fyn, FAK, among others [11–14].

To highlight the diversity of Netrin receptors that have been examined, a new receptor CD146 for Netrin-1 in endothelial cells was recently found to promote angiogenesis [48]. CD146, also known as melanoma cell adhesion molecule (MCAM), member of the immunoglobulin (Ig) superfamily, cooperates in different biological processes including tumor metastasis, lymphocyte activation, and morphogenesis during development, and tissue regeneration. Thus, CD146 plays a critical role in cell proliferation, migration, and tube formation in endothelial cells. Tu and colleagues elegantly demonstrated an interaction between CD146 and Netrin-1. Nevertheless, here we show that HUVEC express low levels of Netrin-1 compared with Netrin-4. We speculate that Netrin-1’s pro-angiogenic contribution rather comes from an adjacent cell present in the stroma, like WJ-MSC, which secrete more Netrin-1 than Netrin-4. Moreover, vascular endothelial cells are in direct contact with blood in the umbilical cord [27]. Hence, it is possible that HUVEC produce more Netrin-4 with anti-angiogenic effect to counteract the Netrin-1 secreted by WJ-MSC. This homeostasis could be imbalanced when the placenta vasculature undergoes dramatic blood vessel remodeling, as in pathological conditions related to pregnancy like IUGR, where Netrin-4 was found with higher expression than Netrin-1. Interestingly, coupled to this disease a derangement of placental vessel formation has been described [27]. It is worth pointing out that diverse researchers have described that placenta can present structural and functional alterations in macrovasculature and microvasculature endothelium and have shown differential response to locally released molecules [53]. Although these effects have been mainly attributed to the expression of classical angiogenic factors such as VEGF or FGF-2 by these cells, our results suggest that the influence of other less studied factors such as Netrin-1 cannot be ruled out. Collectively, our research demonstrates that WJ-MSC produce not only classical angiogenic factors such as VEGF, FGF-2 and EGF [54], but also Netrin-1 as pro-angiogenic regulators of HUVEC. Our results may have implications in the modulation of several pathological conditions related with placental angiogenesis dysfunction.

## Conclusions

Here we demonstrate that WJ-MSC-conditioned medium promotes angiogenesis and that this effect is partially mediated by Netrin-1 and independent of the RhoA/ROCK signaling pathway. This result reveals vital information for clinical approaches aimed at stimulating angiogenic processes. Further in-depth studies to elucidate Netrin-1 mechanism of action will shed light on the development of targeted therapies aiming to restore the physiological balance between Netrin-1, other proteins in the Netrin family and their receptors, for the desired therapeutic effect.

## Additional file

Additional file 1: Figure S1. Differential expression of Netrin-1 in distinct sources of MSC and among passages. **Figure S2.** Conditioned medium (CM) of WJ-MSC promotes endothelial cell migration in vitro. **Figure S3.** Netrin-1 induces angiogenesis in vitro. **Figure S4.** Effect on the angiogenic response after VEGF receptor inhibition on HUVEC. **Table S1.** Sequences for the different primer pairs used for qPCR. Annealing temperature was 60 °C, except for VEGF A that used 55 °C. (DOCX 1630 kb)

## Abbreviations

AD-MSC: Adipose tissue-derived mesenchymal stem cells
BM-MSC: Bone marrow-derived mesenchymal stem cells
CAM assay: Chicken chorioallantoic membrane assay
Col: Collagen I
DCC: Deleted in colorectal cancer
EBM: Endothelial basal media
EGM: Endothelial growth media
FAK: Focal adhesion kinase
FGF-2: Fibroblast growth factor 2
HUVEC: Human umbilical vein endothelial cells
IM: Integra® matrix
MSC: Mesenchymal stromal cells
NTN-1: Netrin-1
NTN-4: Netrin-4
VEGF: Vascular endothelial growth factor
WJ-MSC: Wharton’s jelly-derived mesenchymal stem cells

## References

[1] James JM, Mukouyama YS (2011). Neuronal action on the developing blood vessel pattern. Semin Cell Dev Biol.

[2] Layne K, Ferro A, Passacquale G (2015). Netrin-1 as a novel therapeutic target in cardiovascular disease: to activate or inhibit?. Cardiovasc Res.

[3] Park KW, Crouse D, Lee M, Karnik SK, Sorensen LK, Murphy KJ (2004). The axonal attractant Netrin-1 is an angiogenic factor. Proc Natl Acad Sci U S A.

[4] Nguyen A, Cai H (2006). Netrin-1 induces angiogenesis via a DCC-dependent ERK1/2-eNOS feed-forward mechanism. Proc Natl Acad Sci U S A.

[5] Castets M, Mehlen P (2010). Netrin-1 role in angiogenesis: to be or not to be a pro-angiogenic factor?. Cell Cycle.

[6] Xu K, Wu Z, Renier N, Antipenko A, Tzvetkova-Robev D, Xu Y (2014). Neural migration. Structures of netrin-1 bound to two receptors provide insight into its axon guidance mechanism. Science.

[7] Xie H, Zou L, Zhu J, Yang Y (2011). Effects of netrin-1 and netrin-1 knockdown on human umbilical vein endothelial cells and angiogenesis of rat placenta. Placenta.

[8] Nikolopoulos SN, Giancotti FG (2005). Netrin-integrin signaling in epithelial morphogenesis, axon guidance and vascular patterning. Cell Cycle.

[9] Dakouane-Giudicelli M, Alfaidy N, Bayle P (2011). Tassin de Nonneville A, Studer V, Rozenberg P, et al. Hypoxia-inducible factor 1 controls the expression of the uncoordinated-5-B receptor, but not of netrin-1, in first trimester human placenta. Int J Dev Biol.

[10] Lai Wing Sun K, Correia JP, Kennedy TE (2011). Netrins: versatile extracellular cues with diverse functions. Development.

[11] Rajasekharan S, Baker KA, Horn KE, Jarjour AA, Antel JP, Kennedy TE (2009). Netrin 1 and Dcc regulate oligodendrocyte process branching and membrane extension via Fyn and RhoA. Development.

[12] Shimizu A, Nakayama H, Wang P, König C, Akino T, Sandlund J (2013). Netrin-1 promotes glioblastoma cell invasiveness and angiogenesis by multiple pathways including activation of RhoA, cathepsin B, and cAMP-response element-binding protein. J Biol Chem.

[13] Antoine-Bertrand J, Ghogha A, Luangrath V, Bedford FK, Lamarche-Vane N (2011). The activation of ezrin-radixin-moesin proteins is regulated by netrin-1 through Src kinase and RhoA/Rho kinase activities and mediates netrin-1-induced axon outgrowth. Mol Biol Cell.

[14] Mediero A, Ramkhelawon B, Perez-Aso M, Moore KJ, Cronstein BN (2015). Netrin-1 is a critical autocrine/paracrine factor for osteoclast differentiation. J Bone Miner Res.

[15] Larrivée B, Freitas C, Trombe M, Lv X, Delafarge B, Yuan L (2007). Activation of the UNC5B receptor by Netrin-1 inhibits sprouting angiogenesis. Genes Dev.

[16] Dakouane-Giudicelli M, Duboucher C, Fortemps J, Salama S, Brulé A, Rozenberg P (2012). Identification and localization of netrin-4 and neogenin in human first trimester and term placenta. Placenta.

[17] Dakouane-Giudicelli M, Alfaidy N, de Mazancourt P (2014). Netrins and their roles in placental angiogenesis. Biomed Res Int.

[18] Guo S, DiPietro LA (2010). Factors affecting wound healing. J Dent Res.

[19] Edwards SS, Zavala G, Prieto CP, Elliott M, Martínez S, Egaña JT (2014). Functional analysis reveals angiogenic potential of human mesenchymal stem cells from Wharton’s jelly in dermal regeneration. Angiogenesis.

[20] Ranganath SH, Levy O, Inamdar MS, Karp JM (2012). Harnessing the mesenchymal stem cell secretome for the treatment of cardiovascular disease. Cell Stem Cell.

[21] Shohara R, Yamamoto A, Takikawa S, Iwase A, Hibi H, Kikkawa F (2012). Mesenchymal stromal cells of human umbilical cord Wharton’s jelly accelerate wound healing by paracrine mechanisms. Cytotherapy.

[22] Tran C, Damaser MS (2015). Stem cells as drug delivery methods: application of stem cell secretome for regeneration. Adv Drug Deliv Rev.

[23] Carvalho MM, Teixeira FG, Reis RL, Sousa N, Salgado AJ (2011). Mesenchymal stem cells in the umbilical cord: phenotypic characterization, secretome and applications in central nervous system regenerative medicine. Curr Stem Cell Res Ther.

[24] Kagiwada H, Yashiki T, Ohshima A, Tadokoro M, Nagaya N, Ohgushi H (2008). Human mesenchymal stem cells as a stable source of VEGF-producing cells. J Tissue Eng Regen Med.

[25] Prasanna SJ, Gopalakrishnan D, Shankar SR, Vasandan AB (2010). Pro-inflammatory cytokines, IFNgamma and TNFalpha, influence immune properties of human bone marrow and Wharton jelly mesenchymal stem cells differentially. PLoS One.

[26] Prieto CP, Krause BJ, Quezada C, San Martin R, Sobrevia L, Casanello P (2011). Hypoxia-reduced nitric oxide synthase activity is partially explained by higher arginase-2 activity and cellular redistribution in human umbilical vein endothelium. Placenta.

[27] Boutsikou T, Giotaki M, Gourgiotis D, Boutsikou M, Briana DD, Marmarinos A (2014). Cord blood netrin-1 and −4 concentrations in term pregnancies with normal, restricted and increased fetal growth. J. Matern. Fetal. Neonatal. Med..

[28] Wu W, Tang L (2014). The role of netrin-1 in diabetic retinopathy: a promising therapeutic strategy. Int J Diabetes Clin Res.

[29] Wilson BD, Ii M, Park KW, Suli A, Sorensen LK, Larrieu-Lahargue F (2006). Netrins promote developmental and therapeutic angiogenesis. Science.

[30] Serafini T, Colamarino SA, Leonardo ED, Wang H, Beddington R, Skarnes WC (1996). Netrin-1 is required for commissural axon guidance in the developing vertebrate nervous system. Cell.

[31] Yoneda K, Demitsu T, Nakai K, Moriue T, Ogawa W, Igarashi J (2010). Activation of vascular endothelial growth factor receptor 2 in a cellular model of loricrin keratoderma. J Biol Chem.

[32] Delloye-Bourgeois C, Fitamant J, Paradisi A, Cappellen D, Douc-Rasy S, Raquin MA (2009). Netrin-1 acts as a survival factor for aggressive neuroblastoma. J Exp Med.

[33] Akino T, Han X, Nakayama H, McNeish B, Zurakowski D, Mammoto A (2014). Netrin-1 promotes medulloblastoma cell invasiveness and angiogenesis, and demonstrates elevated expression in tumor tissue and urine of patients with pediatric medulloblastoma. Cancer Res.

[34] Zilberberg L, Shinkaruk S, Lequin O, Rousseau B, Hagedorn M, Costa F (2003). Structure and inhibitory effects on angiogenesis and tumor development of a new vascular endothelial growth inhibitor. J Biol Chem.

[35] Son TW, Yun SP, Yong MS, Seo BN, Ryu JM, Youn HY (2013). Netrin-1 protects hypoxia-induced mitochondrial apoptosis through HSP27 expression via DCC- and integrin α6β4-dependent Akt, GSK-3β, and HSF-1 in mesenchymal stem cells. Cell Death Dis.

[36] Tao H, Han Z, Han ZC, Li Z (2016). Proangiogenic features of mesenchymal stem cells and their therapeutic applications. Stem Cells Int.

[37] Larrieu-Lahargue F, Thomas KR, Li DY (2012). Netrin ligands and receptors: lessons from neurons to the endothelium. Trends Cardiovasc Med.

[38] Castets M, Coissieux MM, Delloye-Bourgeois C, Bernard L, Delcros JG, Bernet A (2009). Inhibition of endothelial cell apoptosis by netrin-1 during angiogenesis. Dev Cell.

[39] Adams RH, Eichmann A (2010). Axon guidance molecules in vascular patterning. Cold Spring Harb Perspect Biol.

[40] Eveno C, Broqueres-You D, Feron JG, Rampanou A, Tijeras-Raballand A, Ropert S (2011). Netrin-4 delays colorectal cancer carcinomatosis by inhibiting tumor angiogenesis. Am J Pathol.

[41] Ke X, Liu C, Wang Y, Ma J, Mao X, Li Q (2016). Netrin-1 promotes mesenchymal stem cell revascularization of limb ischaemia. Diab Vasc Dis Res.

[42] Hsieh JY, Fu YS, Chang SJ, Tsuang YH, Wang HW (2010). Functional module analysis reveals differential osteogenic and stemness potentials in human mesenchymal stem cells from bone marrow and Wharton’s jelly of umbilical cord. Stem Cells Dev.

[43] Maruyama K, Kawasaki T, Hamaguchi M, Hashimoto M, Furu M, et al. Bone-protective Functions of Netrin 1 Protein. J Biol Chem. 2016;291(46):23854–68. 10.1074/jbc.M116.738518 PMC5104911 27681594

[44] Ramkhelawon B, Hennessy EJ, Ménager M, Ray TD, Sheedy FJ, Hutchison S (2014). Netrin-1 promotes adipose tissue macrophage retention and insulin resistance in obesity. Nat Med.

[45] Ke X, Li Q, Xu L, Zhang Y, Li D, Ma J, Mao X (2015). Netrin-1 overexpression in bone marrow mesenchymal stem cells promotes functional recovery in a rat model of peripheral nerve injury. J Biomed Res.

[46] Ke T, Wu Y, Li L, Liu Y, Yao X, Zhang J (2014). Netrin-1 ameliorates myocardial infarction-induced myocardial injury: mechanisms of action in rats and diabetic mice. Hum Gene Ther.

[47] Ding Q, Liao SJ, Yu J. Axon guidance factor netrin-1 and its receptors regulate angiogenesis after cerebral ischemia. Neurosci Bull. 2014;30(4):683–91. 10.1007/s12264-013-1441-9 PMC5562620 24875332

[48] Tu T, Zhang C, Yan H, Luo Y, Kong R, Wen P (2015). CD146 acts as a novel receptor for netrin-1 in promoting angiogenesis and vascular development. Cell Res.

[49] Lejmi E, Leconte L, Pédron-Mazoyer S, Ropert S, Raoul W, Lavalette S (2008). Netrin-4 inhibits angiogenesis via binding to neogenin and recruitment of Unc5B. Proc Natl Acad Sci U S A.

[50] Larrieu-Lahargue F, Welm AL, Thomas KR, Li DY (2011). Netrin-4 activates endothelial integrin {alpha}6{beta}1. Circ Res.

[51] Dakouane-Giudicelli M, Brouillet S, Traboulsi W, Torre A, Vallat G, Si Nacer S (2015). Inhibition of human placental endothelial cell proliferation and angiogenesis by netrin-4. Placenta.

[52] Connolly JO, Simpson N, Hewlett L, Hall A (2002). Rac regulates endothelial morphogenesis and capillary assembly. Mol Biol Cell.

[53] Sobrevia L, Abarzúa F, Nien JK, Salomón C, Westermeier F, Puebla C (2011). Review: Differential placental macrovascular and microvascular endothelial dysfunction in gestational diabetes. Placenta.

[54] Konala VB, Mamidi MK, Bhonde R, Das AK, Pochampally R, Pal R (2016). The current landscape of the mesenchymal stromal cell secretome: a new paradigm for cell-free regeneration. Cytotherapy.

