# Supplementary material for: Netrin-1 acts as a non-canonical angiogenic factor produced by human Wharton’s jelly mesenchymal stem cells (WJ-MSC)
Source: Stem Cell Res Ther. 2017 Feb 28;8:43. doi: 10.1186/s13287-017-0494-5 (PMC5330133; doi:10.1186/s13287-017-0494-5)
Supplement: Additional file 1: Figure S1. — Differential expression of Netrin-1 in distinct sources of MSC and among passages. Figure S2. Conditioned medium (CM) of WJ-MSC promotes endothelial cell migration in vitro. Figure S3. Netrin-1 induces angiogenesis in vitro. Figure S4. Effect on the angiogenic response after VEGF receptor inhibition on HUVEC. Table S1. Sequences for the different primer pairs used for qPCR. Annealing temperature was 60 °C, except for VEGF A that used 55 °C. (DOCX 1630 kb) [file 13287_2017_494_MOESM1_ESM.docx]

**SUPPLEMENTARY FIGURES**


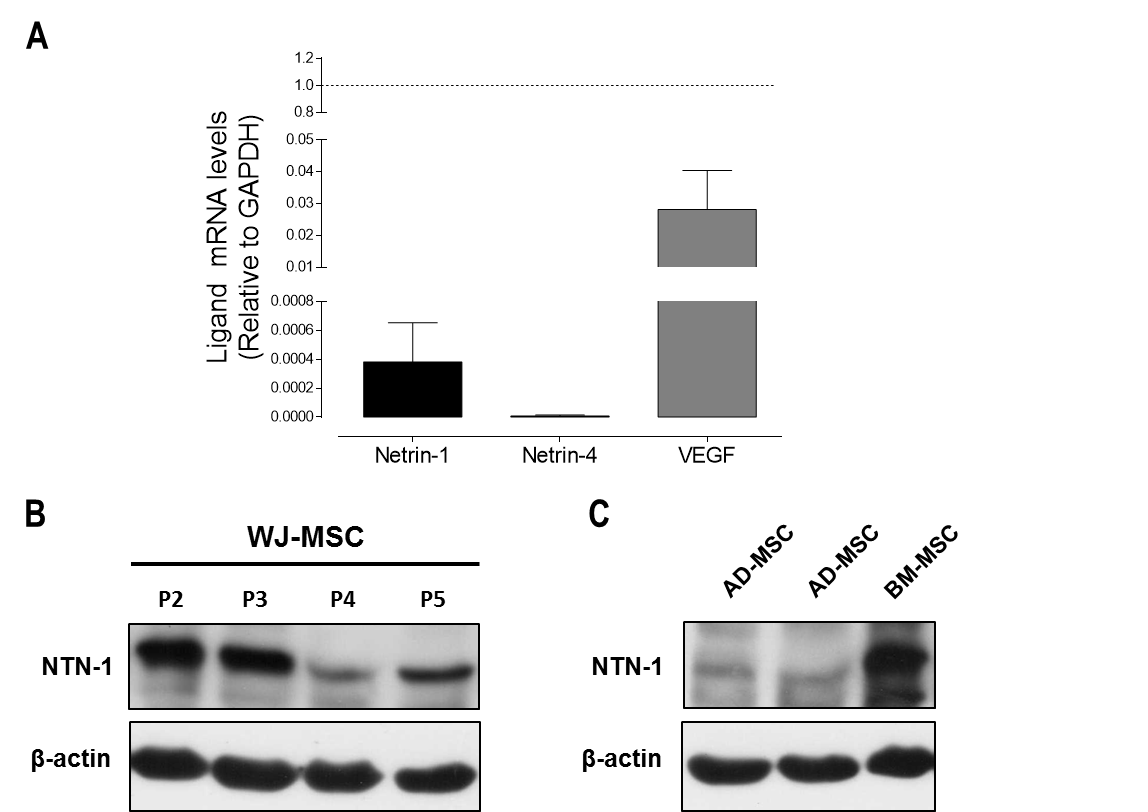


**Figure S1. Differential expression of Netrin-1 in distinct sources of MSC and among passages. *A*.** mRNA levels of Netrins and VEGF were quantified by qPCR relative to GAPDH expression (Dashed line). Values correspond to mean ± S.E.M. (n=5). ***B*.** Western blot for NTN-1, in whole cell lysates from different passages (P2-P5) of the same WJ-MSC culture**.** β-actin was used as internal loading control. ***C*.** Western Blot of NTN-1 expression in whole cell lysate prepared from Human adipose mesenchymal stem cell (AD-MSC, n=2) and Human bone marrow mesenchymal stem cell (BM-MSC, n=1) cultures. β-actin was used as internal loading control.


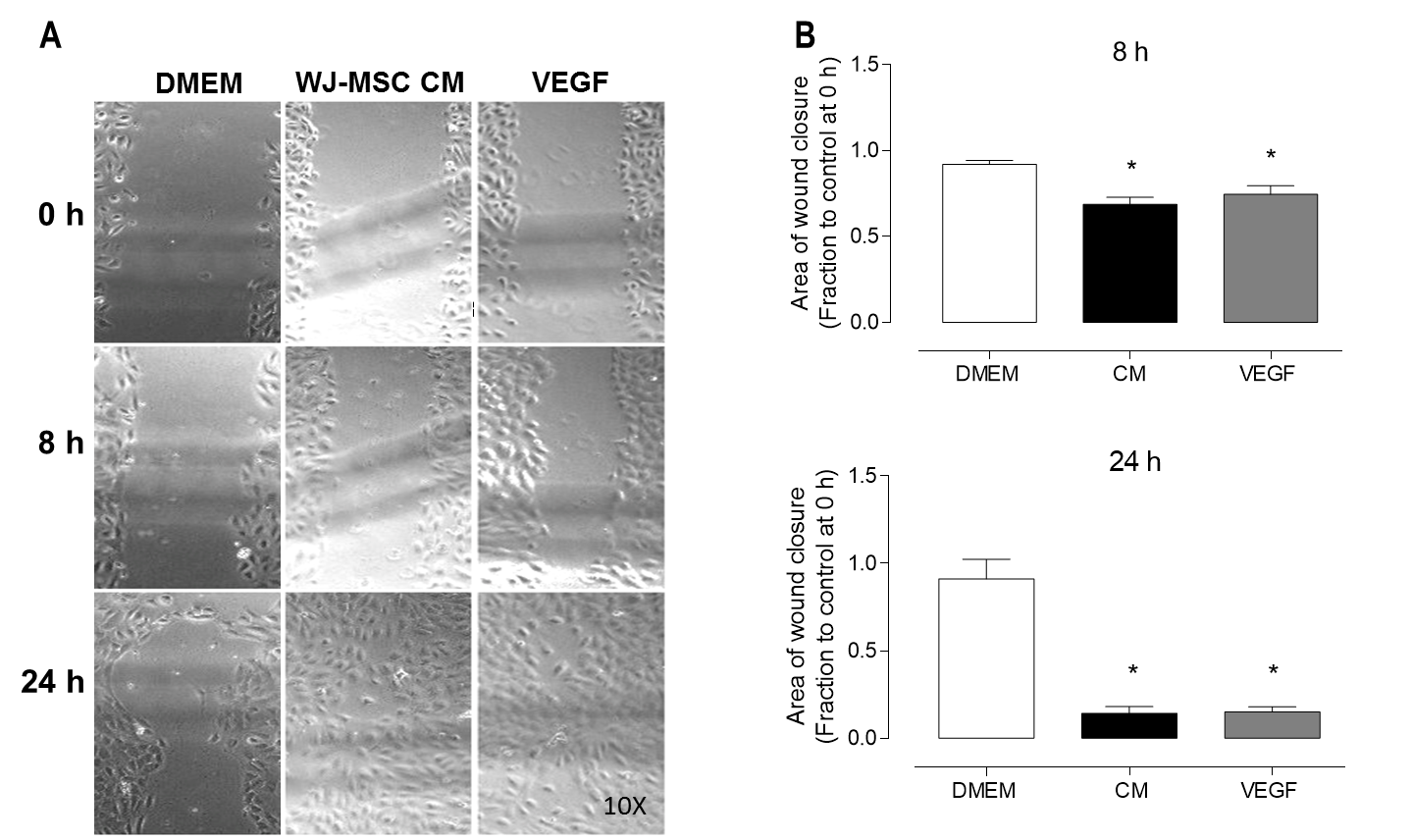


**Figure S2. Conditioned medium (CM) of WJ-MSC promotes endothelial cell migration *in vitro*.** ***A*.** Conditioned media collected for 48 hours from serum starved WJ-MSC cultures were used on endothelial cells to evaluate migration using a scratch assay. DMEM alone was used as internal reference and recombinant human VEGF (40 ng/mL) was used as a positive control. Representative images of each condition are shown (Amplification 10X). ***B*.** Quantified results correspond to the mean ± S.E.M. (n=4, **p* < 0.05).

**
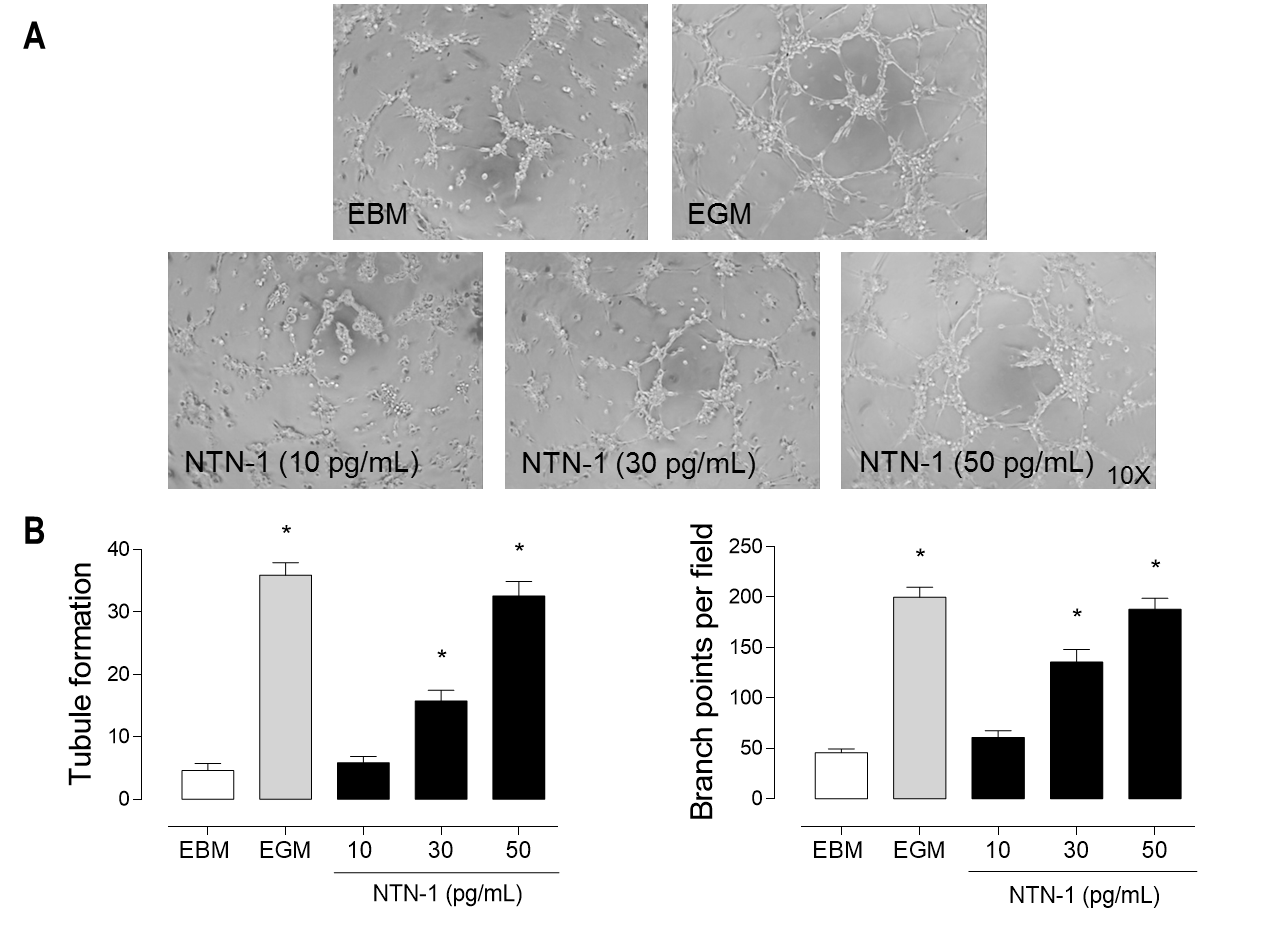
**

**Figure S3. Netrin-1 induces angiogenesis *in vitro*. *A*.** The effect of recombinant human NTN-1 was determined *in vitro* to evaluate angiogenesis through tubule formation assay. Cells were serum-starved and seeded on Matrigel and treated with increased concentrations of NTN-1 for 4 hours. EBM alone was used as internal reference and EGM was used as a positive control. Representative images are shown for each experimental condition (Amplification 10X). ***B*.** Quantified results correspond to the mean ± S.E.M. (n=3, **p* < 0.05 vs. EBM).

**
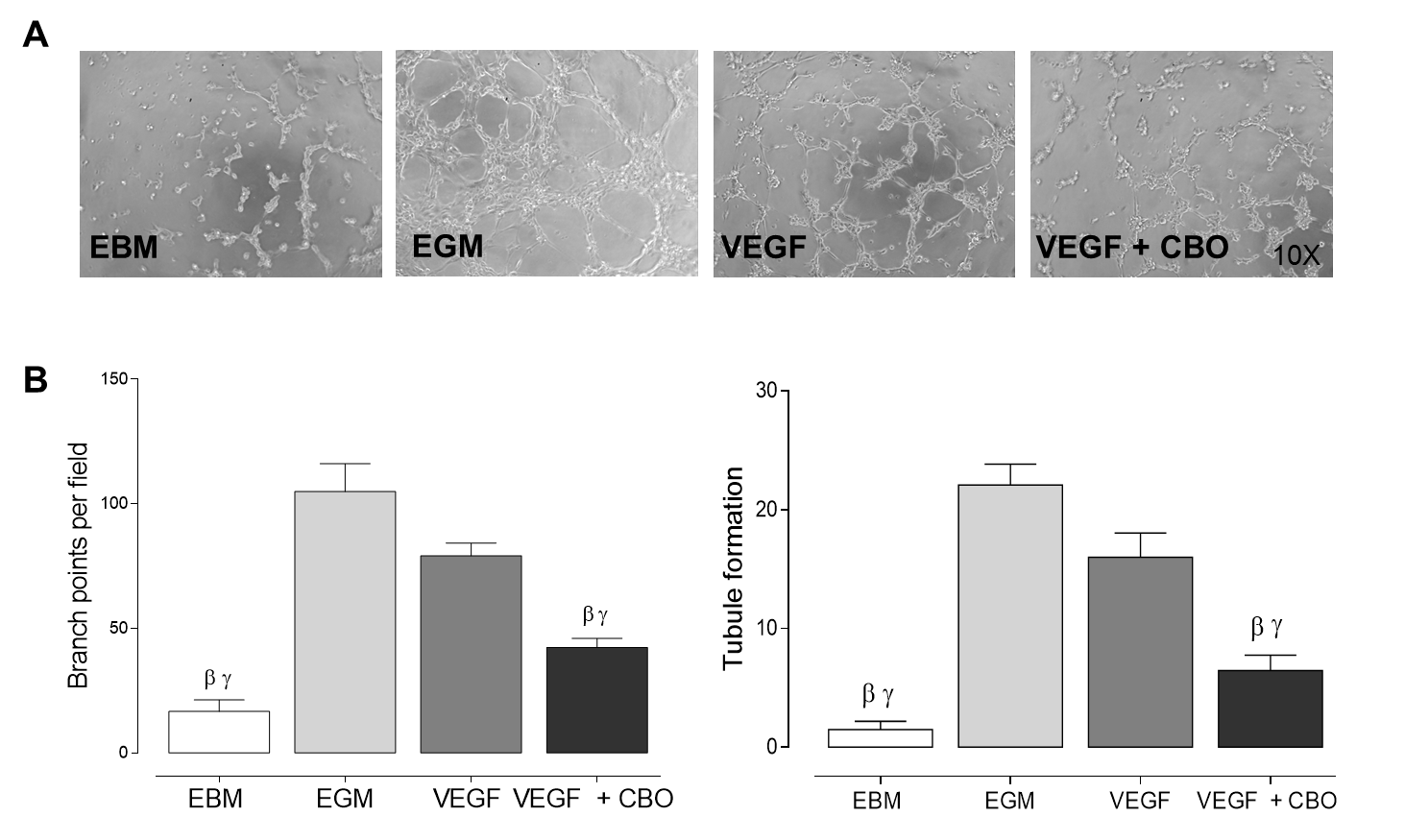
**

**Figure S4. Effect on the angiogenic response after VEGF receptor inhibition on HUVEC. *A*.** Representative images of Matrigel tubule formation and branch points per field of HUVEC exposed 4 hours to EGM, EBM, VEGF (40 ng/mL) in absence or presence of CBO (VEGF-receptor inhibitor, 20 µM). (Amplification 10X) ***B*.** Quantified results correspond to the mean ± S.E.M. (n=3, β*p* < 0.05 vs. EGM; γ*p* < 0.05 vs. VEGF).

| **Target** |  | **Sequence Fw** | **Sequence Rv** |
| --- | --- | --- | --- |
| NTN-1 |  | TGCAAGAAGGACTATGCCGTC | GCTCGTGCCCTGCTTATACAC |
| NTN-4 |  | TCAGCACAACACAGAAGGACA | GGATGGCAGGAACACGGTTTG |
| NEO-1 |  | GCTTCATCAAATTGACGTGGCGGA | AGATGTACACGGTCGCTGGCATTA |
| RGMa |  | ATGGATGGGTATGGGGAGAG | TGCACTTGAGGATCTTGCAC |
| Unc5a |  | TGACTCGTCCATTCTCACCT | CCATTGGTGAGCTGGAACTT |
| DCC |  | GGGGCCACTCTCTGATCCTA | TGCATTTGTCCAATTGGCGG |
| VEGF A |  | CTCTACCTCCACCATGCCAAG | AGACATCCATGAACTTCACCACTTC |
| Integrin α6 |  | ACGTCCATCACGTTAGCTGT | GCATTTGACGTGAGCAGGTA |
| Integrin β1 |  | AGCAACGGACAGATCTGCAA | TGAAGGCTCTGCACTGAACA |
| Integrin β4 |  | CGTCTGGCCTTCAATGTCGT | AGGGTTGTCAACCAGCACTT |

**Table S1.** Sequences for the different primer pairs used for qPCR. Annealing temperature was 60°C, except for VEGF A that used 55ºC.
